# Numerical Study of the Plastic Zone at the Crack Front in Cylindrical Aluminum Specimens Subjected to Tensile Loads

**DOI:** 10.3390/ma16206759

**Published:** 2023-10-19

**Authors:** Lenin Abatta-Jacome, Antonia Lima-Rodriguez, Antonio Gonzalez-Herrera, Jose Manuel Garcia-Manrique

**Affiliations:** 1 Department of Civil Engineering, Materials and Manufacturing, School of Engineering, University of Malaga, 29071 Málaga, Spain; tlima@uma.es (A.L.-R.); agh@uma.es (A.G.-H.); josegmo@uma.es (J.M.G.-M.); 2 Department of Energy Sciences and Mechanics, University of the Armed Forces ESPE, Sangolquí 171103, Ecuador

**Keywords:** fracture, finite element, cylindrical specimen, plastic strain

## Abstract

Cylindrical specimens are of great interest in analyzing mechanical elements’ behavior and investigating phenomena with biaxial loads. It is necessary to identify the behavior of the crack front along the thickness to interpret these results, which are usually based on the hypothesis of a straight crack and the observation of the outer face of the crack front. Based on the work carried out on compact tension type specimens, this work proposes adapting this methodology to cylindrical specimens, adapting the previous finite element models. Cylindrical specimens provide an asymmetric behavior influenced by the radius, where the CT (compact tensile) specimen can be considered the extreme infinite radius case. Combinations of the load level and radius values help us simulate the crack’s behavior under intermediate hypotheses between a plane crack theory and a three-dimensional one. The plastic strain around the crack front will be analyzed as a function of the thickness and the load level applied. The results allow us to validate the numerical methodology and establish the differentiated behaviors of the plastic zones close to the outer and inner radii.

## 1. Introduction

For more than a century, industrialized countries have been researching fatigue issues, with the number of publications related to the subject increasing since 1920 [1]. The complexity of analyzing this phenomenon limits the characterization of fatigue crack propagation based on the classical linear elastic fracture mechanics theory. A broadly studied problem is cylindrical specimens subjected to fracture or fatigue processes. These specimens present behaviors similar to those of elements such as tubular structures or pipes of interest in industrial applications [2].

This geometry enables the study of biaxial issues. However, to correctly interpret biaxial problems, precisely knowing the specimen’s mode I behavior is necessary. Crack propagation is related to the local stresses and strains occurring within the surface and the specimen. The traditional experimental method allows an accurate characterization exclusive of the surface of the specimen. However, the inner information of the specimen is limited [3]. Based on its evolution, correlations are made with crack growth variables.

This involves the assumption that the crack grows straight. However, in previous work on CT specimens, theoretically characterized by generating straight cracks, it was already detected that they evolved towards curved shapes due to the different growth rates along the thickness. The thickness and load level are the fundamental parameters that characterize this behavior. This results in a different distribution of the stress intensity factor along the thickness, differences in the plasticized volume around crack front areas, and, consequently, differentiated fatigue crack-closing effects. Previous works have extensively studied the interaction between all these factors [3,4,5,6,7,8,9,10,11,12,13,14,15,16,17,18,19,20,21,22,23,24,25].

Therefore, it is necessary, and the main objective of this work, to carry out a preliminary study in mode I of cylindrical specimens to validate previous numerical methodologies that allow future numerical approximations to the biaxial problem. The numerical work methodology applied to the CT specimens will be transferred for this study. It represents an increase in complexity since, in addition to the thickness, the radius of curvature becomes an additional geometric element with a high influence on the results. Variations in these parameters allow us to analyze intermediate cases between extreme cases such as theoretical straight cracks (infinite radius as CT specimen behavior) and thick tridimensional specimens (small radii relative to the thickness). Numerical methods have helped with the study of fatigue and fracture problems. Their use has become widespread and covered more case studies as computational means have developed, particularly the finite element method [26,27,28,29,30,31,32,33]. The limiting factor found is the high computational cost due to the discretization needed [4,5,7].

In areas close to the crack, the size of the area where the plastic strain occurs requires a large number of elements for its finite element analysis, which directly influences the results of stress and strain on the crack front [4,5,7]. However, the discretization at the crack has been determined through established theoretical criteria, such as the Dugdale plastic zone radius [22]. The use of specific meshing strategies for these problems has been extended, with a first volume in the crack environment, fine and controlled meshing, and another automatic one where the result gradients do not present convergence problems for the accuracy of the results [5].

Research with a higher discretization level and computational cost has studied the concept of crack tip closure, determining that the discretization level modifies the relationships between the crack tip parameters [16].

Other numerical research has studied the propagation of cracks in microcrystalline structures based on propagation through remeshing, evaluating the direction of growth by applying a damage indicator based on the plastic accumulation at the crack tip, emphasizing the contribution of shear and normal stresses that act in the slip planes, and demonstrating with diffraction tomographic techniques that the crack propagates mainly in the crystallographic planes [34]. Other studies have defined design criteria that start from the fatigue crack propagation threshold. It is a crucial parameter within the framework of a damage tolerance analysis that can significantly affect the residual useful life of the component until failure [35]. Furthermore, the effects have been determined through the thickness in the vicinity of the crack since there is a singularity in the vertex near the surface, determining the importance of ultrafine discretization [7]. The plastic deformation area around the crack tip has not only been evaluated numerically; it has also been experimentally studied using Vickers micro-identification methods [36] and digital image measurements to visualize the evolution of the plastic zone [37,38,39].

Many experimental works have also focused on the fatigue analysis of different specimens. Crack propagation has been investigated in circular components made of ST-52-3N carbon steel, where image correlation has contributed to assessing propagation through the application of tensile/torsional loads [40].

2050 T8 aluminum was tested using a universal testing machine with a sinusoidal load function at 0.2 Hz (ASTM E647) [41]; the maximum stress applied was 80 MPa, and a load ratio of 0.1 determined that closure crack growth was a primary mechanism for the fatigue crack’s growth [42]. The behavior of flat plates with stress concentrators has been evaluated using the modified Paris Law, determining the fatigue life of a component [43]. The propagation of cracks due to internal defects of the test piece has been analyzed, and it was found that they do not have significant effects during the component’s life [44]. Recent research has concluded that the influence of crack closure on fatigue crack growth rates may be significantly less when modeled using the damage accumulation principle [45].

Numerical models have been compared with experimental ones based on the stress singularity exponent as a control parameter and a second parameter using the stress intensity factor. For this purpose, specimens with a notch on a single front were studied, as in CT specimens when cyclic loads are applied. Specimens of 7075 aluminum and EA4T steel with various thicknesses were used. Cyclic loads were applied by measuring the curvature angles at the fatigue crack front. The experimental methodology was compared with the numerical one, providing comparable results [46]. Other types of research have been carried out, such as a numerical study of fatigue in metallic materials in a vacuum, which determined similar behaviors in terms of the conceptual basis of fatigue [47]. In 2020, research on crack closure induced using laser blasting was carried out, determining that a previous crack had a minimal effect on the residual stress field compared to a standard specimen [48].

In previous works [4,6,8,49], a finite element analysis methodology has been developed to study the characteristic parameters of mode I fracture and fatigue problems with specimens with a two-dimensional theoretical behavior, such as aluminum compact tensile (CT) specimens. Mesh density strategies were established for the area surrounding the crack front and for the thickness, the plastic regions, the influence of the plastic wake on the crack closure, and its relationships with the stress intensity factor. One of the common conclusions is that there are three-dimensional response phenomena along the thickness and that they influence the curvature of the crack front.

Cylindrical specimens provide an asymmetric behavior where combinations of the load level, radius values, and thickness shed light on the behavior of the crack when comparing the exterior and interior free surfaces. In the present paper, we will focus on studying the plasticized area. This is a very representative parameter of the state of the crack front. The results obtained will be compared with the CT-type specimens.

This research work is structured as follows: The following section briefly describes and justifies the methodology. Subsequently, the results obtained are presented, an extensive analysis is reported, and, finally, the main conclusions are described.

## 2. Materials and Methods

**Figure 1:**
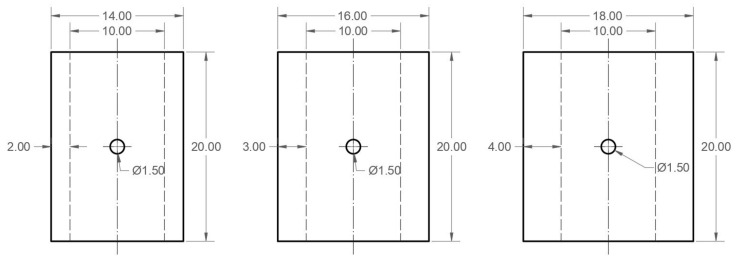

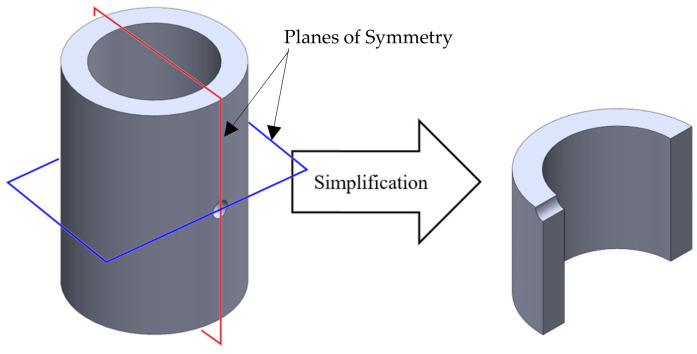
Geometry and simplification of the cylindrical specimen, dimensions in millimeters.

This section deals with the technical parameters of the numerical model. This research was developed based on Al-2024-T351 aluminum specimens with a cylindrical geometry of 2, 3, and 4 mm in wall thickness. An internal diameter of 10 mm and a stress concentrator perpendicular to the load axis of 1.5 mm in diameter was used, similar to the geometry proposed by Mokhtarishirazabad in 2017 [40]. Simplifications were applied due to the geometric symmetry condition, as shown in Figure 1.

**Figure 2:**
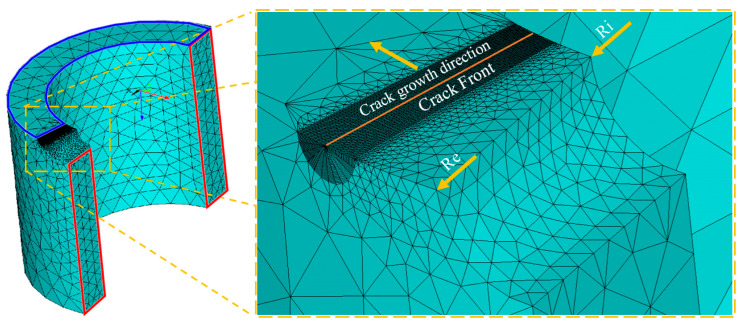
Finite element model.

The cylindrical specimen was modeled three-dimensionally, evaluating the plastic behavior of the material. Figure 2 shows how the geometry and the associated discretization were created using ANSYS APDL programming. In this research, the growth of cracks was not analyzed. Based on a previous crack length, the plastic area and other parameters were evaluated in fracture simulation.

The area with the highest mesh density corresponds to the crack front. The most significant gradients in the results of the tensile stresses and strains are produced within this area. It is known that this is the most critical region of the model. The size of the elements around the crack tip must be accurately analyzed to prevent losing information on the phenomenon studied.

The specimen was divided into two clearly defined volumes for this purpose. The first was the crack front, where a homogeneous, uniform, and undistorted mesh was made using hexahedral elements, while the second was formed with tetrahedral elements. This allowed a transition from this region to the rest of the specimen. These two volumes avoided penalizing the model by reducing the computational cost.

Figure 2 shows the differences in the element size between both regions. The coordinate origin is at the center of the cylindrical specimen’s internal radius (Ri). The *x*–*y* plane is in the plane of the crack front. The *z*–axis is in the direction of the specimen’s extrusion. In the critical zone of the crack front, the minimum element size was defined based on the size of the plastic zone, for which the Dugdale expression [22], Equation (1), was used:

(1) $$r_{pD}=\frac{\pi }{8\alpha }{\left(\frac{K_{I}}{\sigma_{y}}\right)}^{2}$$

where $K_{I}$ is the stress intensity factor and α is a restraining factor. The value of 1 was taken according to the conclusions of previous works on CT specimens [4,5,7]. The element size around the crack tip was adjusted through an iterative process based on a convergence analysis, achieving a positive balance between the precision and the computational cost.

A cone methodology was used to mesh the critical zone, where the size of the final element of the semi-circles was *π*/60 of the $r_{pD}$. The radius of the semi-circumference corresponded to 1/45 of the *r_pD_* before and after the crack advance in the direction perpendicular to the crack advance. Finally, the element’s size was 1/120 of the thickness in the direction parallel to the crack front.

These values are consistent with those obtained in the convergence analysis of previous works for CT-type flat specimens [4]. The mechanical properties considered for the numerical simulation were those of aluminum alloy Al-2024-T351 [50]: modulus of elasticity E = 73 GPa, yield stress $\sigma_{y}$ = 325 MPa, Poisson’s ratio µ = 0.33, and an elastoplastic behavior with isotropic hardening. The results presented in this research were calculated based on the ratio H/E = 0.003, which adjusts to the real behavior of the aluminum alloy under analysis. H is the elastoplastic behavior of isotropic hardening. A quarter of the total geometry was generated, and the respective symmetry conditions were applied to the model. In Figure 1 and Figure 2, the planes of symmetry are identified. In the areas coinciding with these planes, a zero-displacement condition was imposed on all nodes in the direction perpendicular to the plane of symmetry. In the extreme area of the semi-cylinder, the load distributed in the longitudinal direction of the specimen (*z*-axis) was applied, and displacements in the transverse plane were restricted (*x* and *y* axes).

For comparative purposes, all the models were developed based on the stress intensity factor $K_{I}$ considering that it is a through-crack for a plate of finite length, for which the following expression was, Equation (2):

(2) $$K_{I}=Y\times \sigma \times \sqrt{\pi \times a}$$

where Y is a geometry-dependent parameter, a is the length of the half-crack, and σ is the normal tensile stress. Table 1 shows the values of $K_{I}$ used in the models, as well as their load equivalence in Newtons. These values were chosen to cover a significant range of fatigue behavior for this material.

For post-processing, MATLAB R2023a was used based on the ANSYS APDL results dataset, including node coordinates, displacements, strains, stresses, and others. All these computations were conducted on a Dell Workstation 7710 computer.

## 3. Results and Discussion

Several numerical simulations were carried out to analyze the variations in the different parameters studied. It has also been verified that the stress and strain fields were consistent with previous investigations. This section presents a selection of the results obtained, focusing mainly on the plastic region around the crack front.

### 3.1. Plastic Zone Size

**Figure 3:**
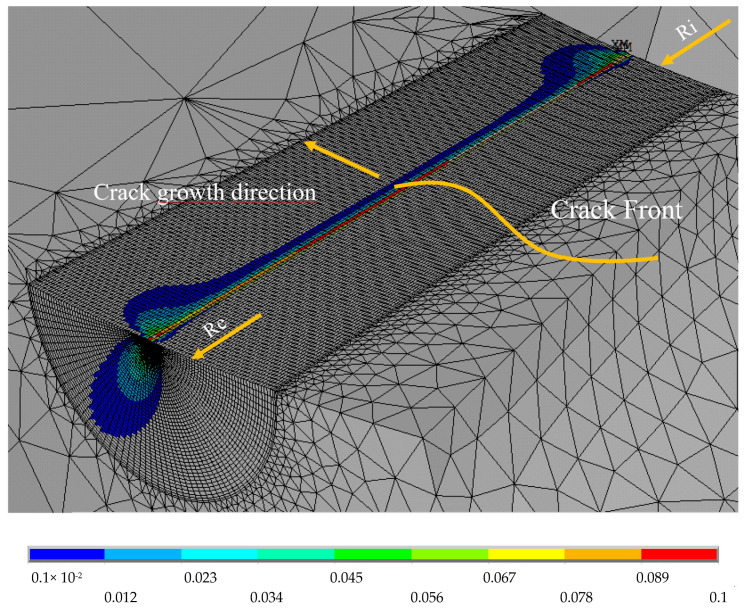
Plastic zone of the crack.

The plastic zone close to the crack front was shown accurately. The plastic zone plays an essential role in the mechanical behavior of a component [51]. Figure 3 shows the area of plastic analysis and illustrates the behavior of plastic strain in the crack along the thickness.

Figure 3 presents the nodal results of the plastic deformation of the specimen, excluding the regions where no plasticization was achieved. If the figure is observed, a behavior different from the theoretical “bone shape” can be identified in the regions close to the outer faces of the specimen. These results were related to the cylindrical specimen’s internal radii, Ri, and external radius, Re.

**Figure 4:**
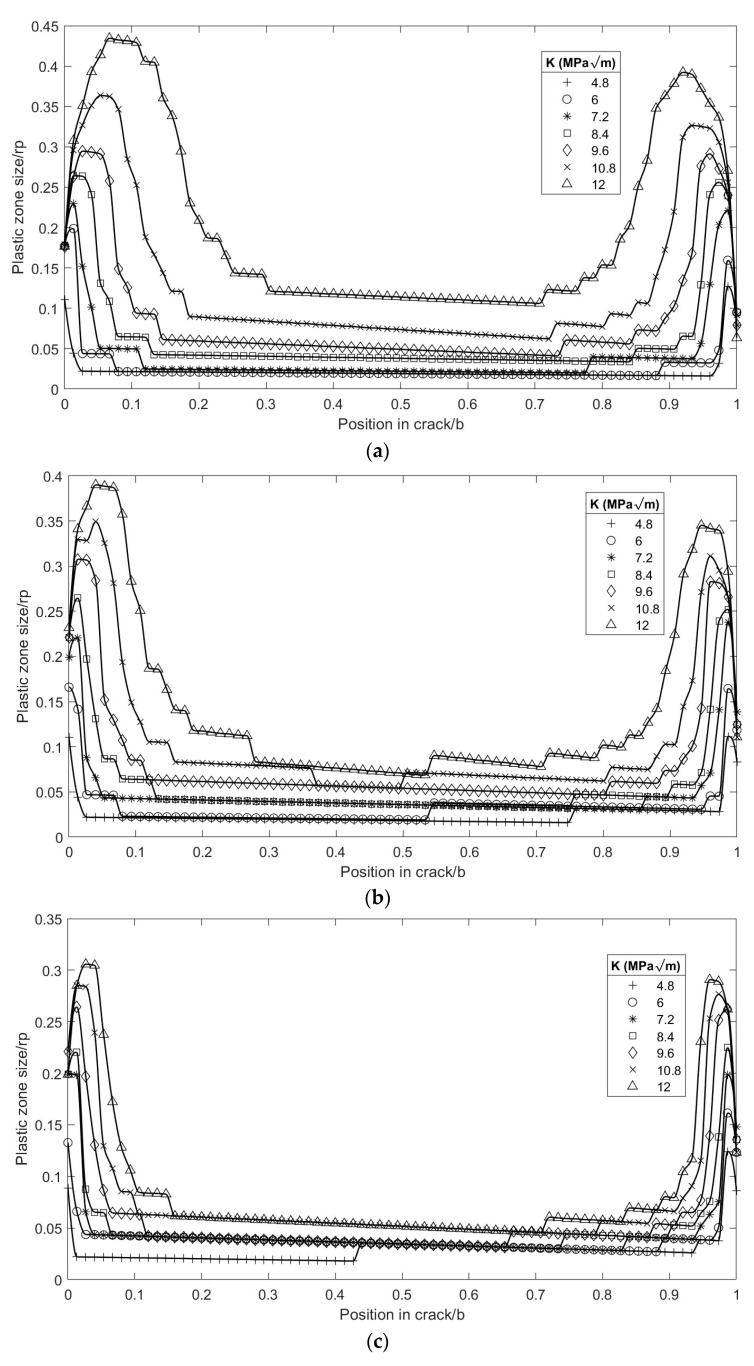
Evolution of the plastic zone in the plane of the normalized crack front. Re = 0; Ri = 1; specimen thickness: (**a**) 2 mm, (**b**) 3 mm and (**c**) 4 mm.

The results obtained by varying the geometry (specimen thickness) and load levels (K) were compared to evaluate the variation in the plastic strain in the plane of the crack along the thickness.The normalized plastic strain in specimens of 2, 3, and 4 mm thickness under different load values is shown in Figure 4, where the left side corresponds to the external radius Re and the right to the internal radius Ri. The plastic strain in the central part had an increasing tendency towards the external radius, marking a difference from the flat specimens, which showed a constant, non-increasing plastic strain size [4]. There was a significant increase in the size of the normalized plastic strain both in the internal and external radii, starting from the midpoint and considering the direction towards the external surface. It is also shown that, near the surface, the trend was reversed, and the plastic strain size decreased dramatically, as expected.

**Figure 5:**
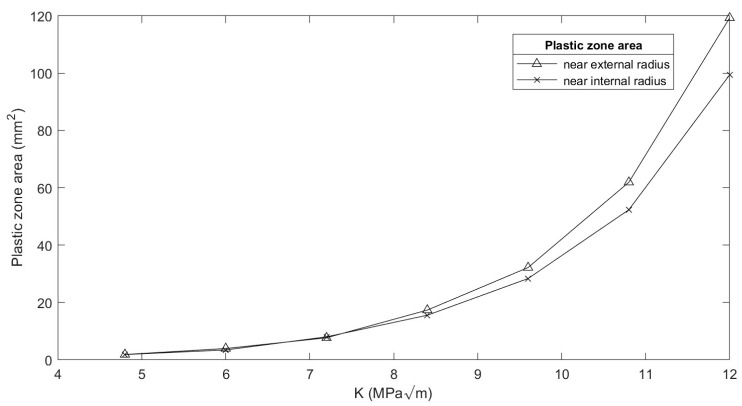
Comparison of areas at the extremes of plastic strain associated with Ri and Re; specimen thickness: 2 mm.

The stress intensity factor and its influence on the curvature of the crack front were evaluated. For the distribution of the plastic zone, stress intensity factor values between K = 4.8 and 12 MPa·m^1/2^ were used along with different thicknesses of specimens (2, 3, and 4 mm). It was found that increases in the load level cause an extension to the plastic strain zone towards the specimen’s interior. All these results were consistent with those obtained with the CT specimen [4]. An important parameter to compare is the variation in areas corresponding to plastic strain in the plane of the crack front at the borders, associated with the Ri and Re. For a quantitative analysis, the plasticized area in each region was evaluated, in terms of the half close to the external face (0 to 0.5 in Figure 4 x-axis) and the half close to the internal face (0.5 to 1 in Figure 4 x-axis). Figure 5 shows the quantitative variation in these areas as a function of the applied load for a 2 mm thick specimen. It was observed that the sizes of both plastic regions increased with the load level, as expected. At low load levels, the plastic strain had a significant geometric similarity for the same load level both outside and inside the specimen. However, as the level of $K_{I}$ increased, the differences between the plasticized areas inside and outside the specimen appeared and were accentuated, as shown in Figure 5.

**Figure 6:**
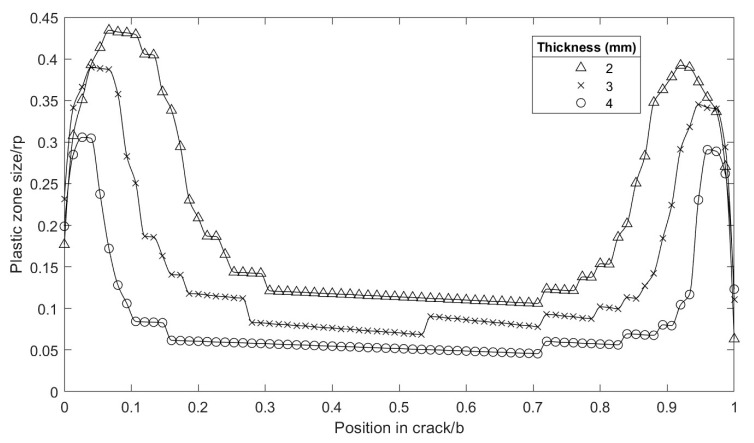
Comparison of the plastic zone in different thicknesses; load K = 12 MPa·m^1/2^; normalized *x*-axis.

Another interesting analysis point was to evaluate the plastic strain under the same load regime for different thicknesses (see Figure 6). The magnitude of the plastic zone in the crack front plane increased when the thickness of the specimen decreased. This was because the global normal stress increased due to the decrease in the resistant section. At a distance of 0.5 of the thickness, the magnitude changed from 0.05 $r_{pD}$ to 0.1 $r_{pD}$, for thicknesses of 4 and 2 mm, respectively.

**Figure 7:**
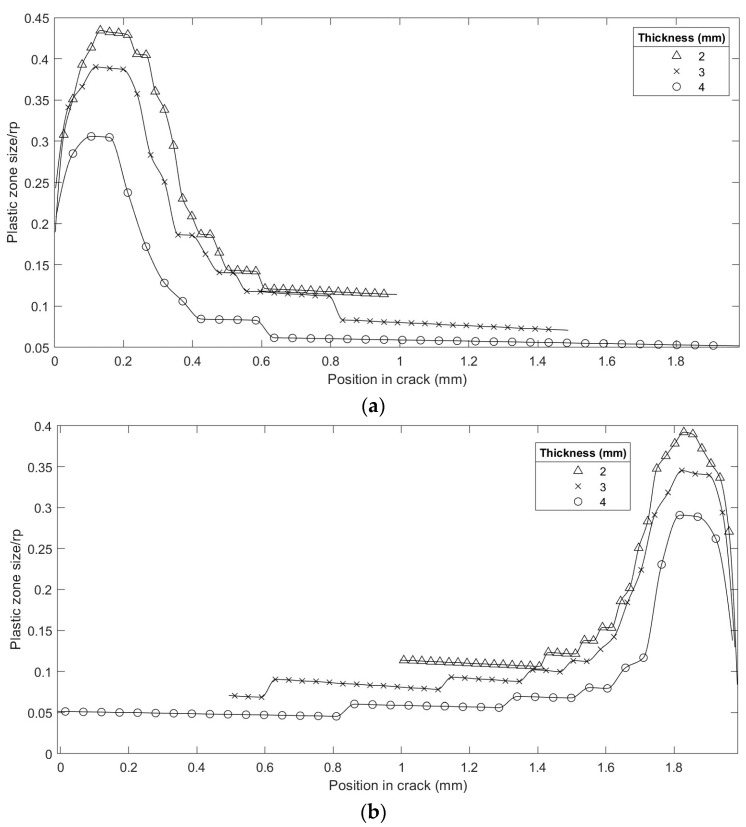
Comparison of the plastic zone in different thicknesses in absolute magnitude; load = 12 MPa·m^1/2^; (**a**) near the external radius; (**b**) near the internal radius.

Figure 7 shows the plastic zone’s absolute magnitude under the same load level. It was observed that the geometry of the plastic strain was similar in the area close to the external surface, both for the external radius (Figure 7a) and the internal radius (Figure 7b).

**Figure 8:**
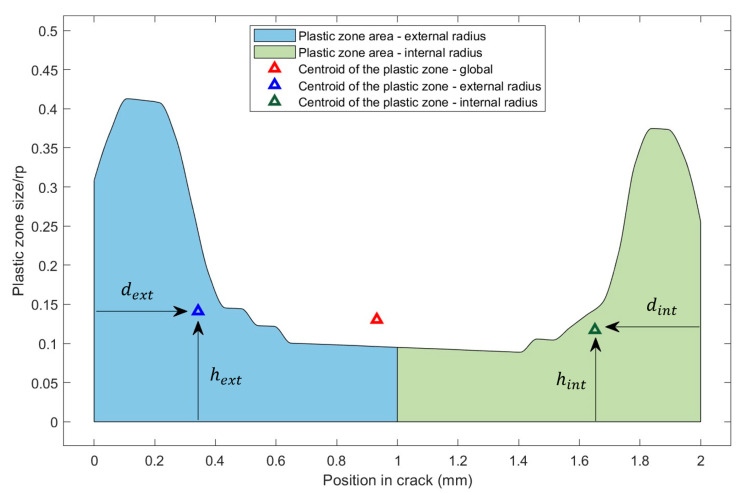
Location of the global center of gravity; load = 12 MPa·m^1/2^; specimen thickness: 2 mm.

The present results have been quantified to evaluate the different parameters. A cylindrical specimen with a wall thickness of 2 mm was considered with a $K_{I}$ = 12 MPa·m^1/2^, and we analyzed the position of the center of gravity of the plastic strain in the flat area of the crack front. Figure 8 shows that the global center of gravity was displaced towards the external radius Re. This behavior seems to be a characteristic property of the cylindrical geometry. In flat specimens, the centroid coincides with the center of the specimen’s thickness, as represented by Camas [4], who evaluated the dependence of parameters concerning the center of gravity based on the area of size generated in the plane of plastic strain in CT specimens. The plastic zone in Figure 8 was divided to half the thickness. The position of the center of gravity was calculated from the external fronts represented by $d_{ext}$ and $d_{int}$. Based on their positions, it was identified that the distances of the centers of gravity associated with the Re and Ri showed characteristics typical of cylindrical specimens, a phenomenon that does not occur in flat specimens.

**Figure 9:**
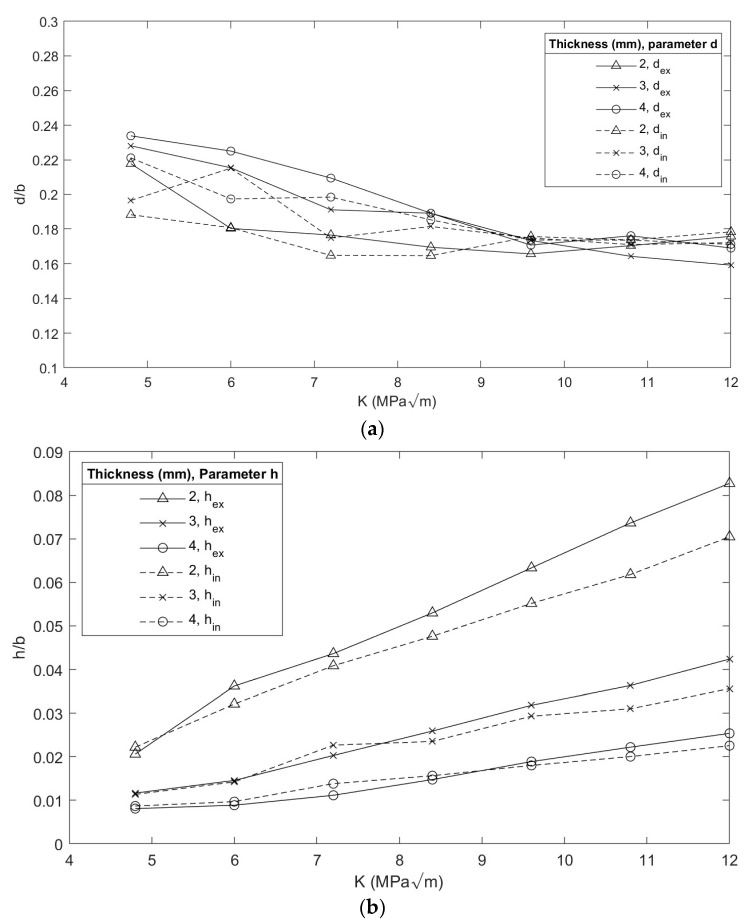
Evolution of gravity center position of the plastic zone: (**a**) with respect to $d_{ext}$ and $d_{int}$, (**b**) with respect to $h_{ext}$ and $h_{int}$.

Figure 9a shows that the increase in load implied a displacement of the center of gravity towards the surface of the specimen due to the extension of the plastic zone in this area, in a similar condition to that established by Camas [3]. Furthermore, the distance of the center of gravity concerning the crack front was evaluated, in terms of $h_{ext}$ and $h_{int}$ (Figure 9b). It was identified that, based on the increase in load, the center of gravity moved away from the crack front due to the increase in the plastic zone. A plastic zone of greater magnitude associated with the external radius was identified, characteristic of the cylindrical specimens.

### 3.2. Stress State along the Thickness

**Figure 10:**
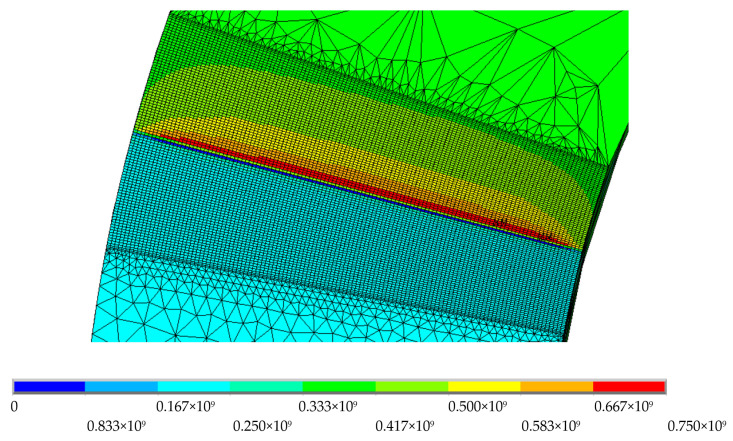
Normal stress σz (Pa) in the plane of the crack front in the cylindrical specimen.

The normal stress state was evaluated in the σz direction, parallel to the load axis, with a $K_{I}$ of 12 MPa·m^1/2^ and a thickness of 2 mm (Figure 10). It was identified that the highest values of normal stress in the *z*-axis were shown at the crack front. Differences were found in the normal stress values along the crack edge. This variation was associated with the specimen’s internal and external radius geometry.

**Figure 11:**
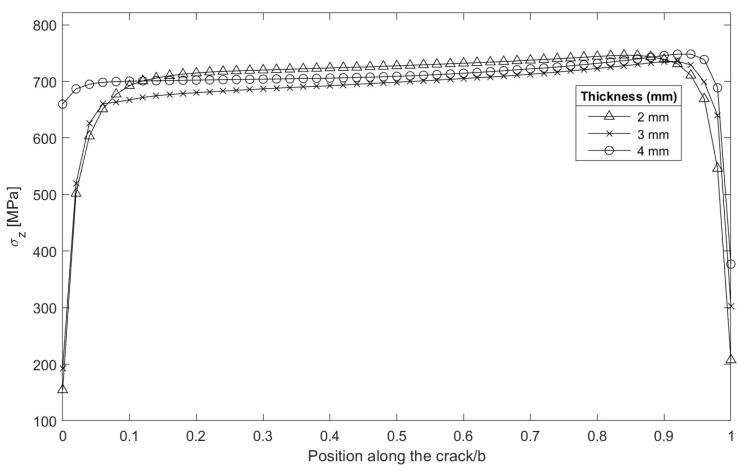
Normal stress σz in the plane of the crack front; load = 12 MPa·m^1/2^; the origin of coordinates (zero) corresponds to the external radius of the specimen.

Figure 11 shows the normalized transition of the normal stress state parallel to the load axis for thicknesses of 2, 3, and 4 mm and a $K_{I}$ of 12 MPa·m^1/2^. The transition between plastic zones was evaluated to observe the distribution from shapes similar to plane strain to plane stress at the crack front. Very close to the external surface, the value tended to zero. The geometric trend of the evolution of the normal stress analyzed in the plane along the crack front was greater in the internal radius, contrary to the slopes presented when the plastic strain was analyzed. The σz evolution in each case present the same positive slope from external to internal face. This is characteristic of cylindrical specimens. In flat CT specimens, σz evolution through thickness has a constant trend in its central band [4].

### 3.3. Numerical Verification

**Figure 12:**
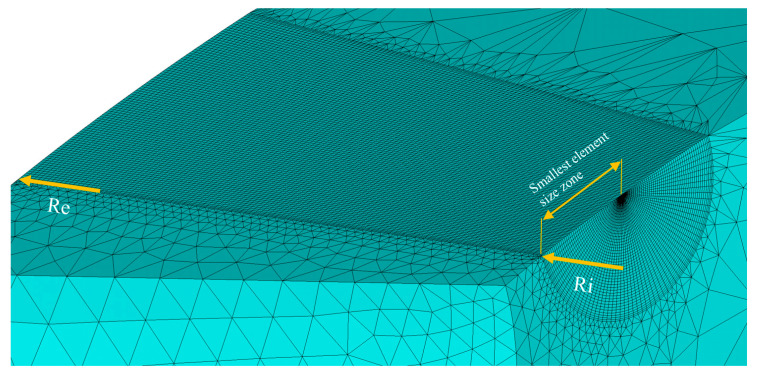
Discretization around the crack tip, element size analysis zone.

The element size at the crack front is a crucial parameter. In order to ensure the quality of the results, an analysis of the element size was carried out to validate the findings obtained in this research. In the methodology, the type of elements used to generate the numerical model and their geometric relationship concerning the *r_pD_* of the specimen were detailed. For this analysis, the geometric relationship that produced the smallest element size was considered. This was presented in the radius of the internal semicircle used in the cone methodology, which had 45 divisions (Figure 12); all of this was performed in search of a computational cost balance and to improve the definition of results.

**Figure 13:**
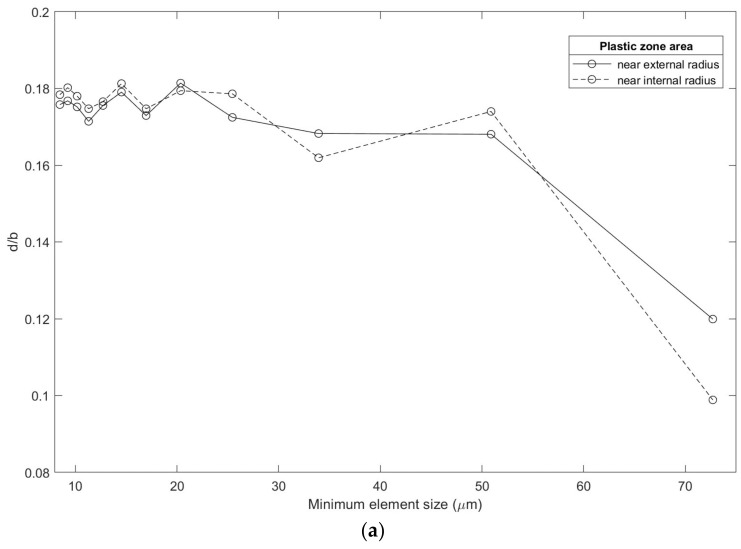

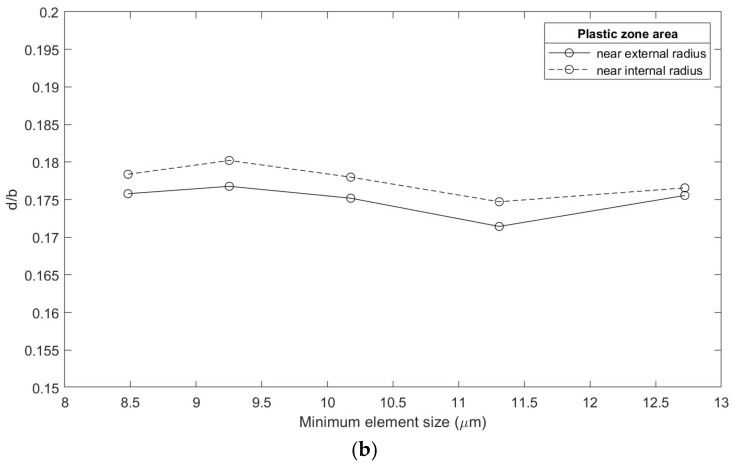
Finite element size analysis: (**a**) convergence analysis, (**b**) ultrafine finite element analysis; the vertical axis is normalized.

Through several iterations, the evolution of the normalized parameter with the minimum element size used in the numerical model was evaluated. In Figure 13a, a convergence graph is shown. As the element size increased, the stabilization of results was lost. When the size of the elements varied between 8.5 µm and 13 µm, stable results were shown in a similar condition with related research works (Figure 13b) [4,5].

A marked tendency was shown in the convergence of the plastic strain at the crack front. In order to obtain correct results, it is essential to specify that the plastic strain must be contained within the $r_{pD}$. The classic “bone shape” plastic zone was distorted when increasing the elements’ size. In this research, the smallest element size was used, considering multiple iterations, in order to maintain a balance with the computational cost, and to achieve a high definition in the results of interest. Using an ultrafine mesh to describe the phenomenon adequately was incredibly convenient. However, the results are very sensitive to the model’s response around the crack tip, as established in [4].

## 4. Conclusions

A numerical analysis was carried out using finite elements of a cylindrical specimen subjected to tensile loads on its axis. The analysis of the results focused on the plastic zone near the crack front, where the calculation method developed in previous works for compact tension (CT) test specimens has been validated. These findings allow the study of various behaviors on the crack front of cylindrical samples of different thicknesses and loads. The following are the findings of the numerical study:

- When evaluating the shape of the plastic zone in the flat zone of the crack front in cylindrical specimens, a clear difference was found concerning flat specimens. The geometry of its ends showed a quantifiable geometric difference in area parameters associated with the internal and external radii. This difference increased as the load values increased. For low load values, their geometric characteristics tended to be similar;
- It was found that, in the plane of the crack front, there was a greater plastic strain associated with the external radius;
- It was identified that the plastic strain was not constant in the central part as in flat specimens. It maintained an increasing trend towards the external radius of the cylindrical specimen;
- The centroid of the plastic strain presented in the flat area of the crack front was not located in the central part, as in flat specimens. The centroid showed a clear trend towards the external radius;
- A clear geometric trend of the plastic strain presented in the flat zone of the crack front was found when evaluating qualitatively with non-normalized thickness parameters under the same load regime;
- The evolution of the plastic strain’s size in the crack front’s flat area depended largely on the thickness and the load applied for the analysis. However, this strain maintained its clear relationship with the geometry of the cylindrical specimen, that is, it was associated with the external and internal radii;
- The normal stress σz at the crack front showed a trend with a positive slope in the central band, a characteristic parameter of cylindrical specimens.

## Figures and Tables

**Table 1:** Stress intensity factor and load applied to specimens.

$K_{I}$ $\left[Mpa\sqrt{m}\right]$	Load [N] Thickness 2 mm	Load [N] Thickness 3 mm	Load [N] Thickness 4 mm
1.20	594.51	903.68	1223.38
2.40	1189.02	1807.36	2446.76
3.60	1783.53	2711.04	3670.14
4.80	2378.04	3614.72	4893.52
6.00	2972.55	4518.40	6116.90
7.20	3567.06	5422.08	7340.28
8.40	4161.57	6325.76	8563.66
9.60	4756.08	7229.44	9787.04
10.80	5350.59	8133.12	11010.42
12.00	5945.10	9036.80	12233.80

## Data Availability

Not applicable.

## Nomenclature

a	Crack length.
b	Specimen thickness.
$d_{int}$	Distance to the exterior face of the center of gravity of the plastic size area, associated with the internal radius.
$d_{ext}$	Distance to the exterior face of the center of gravity of the plastic size area, associated with the external radius.
E	Modulus of elasticity.
$K_{I}$	Stress intensity factor, mode I.
H	Elastoplastic behavior with isotropic hardening.
$h_{int}$	Distance from the crack front to the center of gravity, associated with the internal radius.
$h_{ext}$	Distance from the crack front to the center of gravity, associated with the external radius.
Nu	Poisson’s Ratio.
Re	Internal radius
Ri	External radius
$r_{p}$	Calculated plastic radius.
$r_{pD}$	Dugdale’s plastic zone size.
Y	Geometric parameter, associated with the stress intensity factor.
α	Restriction factor, associated with Dugdale’s plastic radius.
σ	Normal tensile stress.
$\sigma_{y}$	Yield strength.

## References

[1] Schütz W. (1996). A history of fatigue. Eng. Fract. Mech..

[2] Jasinski J.J., Tagowski M. (2022). FEM Simulation of the Riveting Process and Structural Analysis of Low-Carbon Steel Tubular Rivets Fracture. Materials.

[3] Garcia-Manrique J., Camas D., Parrón-Rubio M., Gonzalez-Herrera A. (2018). Corrections in numerical methodology to evaluate plasticity induced crack closure along the thickness. Theor. Appl. Fract. Mech..

[4] Camas D., Garcia-Manrique J., Gonzalez-Herrera A. (2011). Numerical study of the thickness transition in bi-dimensional specimen cracks. Int. J. Fatigue.

[5] Camas D., Garcia-Manrique J., Gonzalez-Herrera A. (2012). Crack front curvature: Influence and effects on the crack tip fields in bi-dimensional specimens. Int. J. Fatigue.

[6] Garcia-Manrique J., Camas D., Lopez-Crespo P., Gonzalez-Herrera A. (2013). Stress intensity factor analysis of through thickness effects. Int. J. Fatigue.

[7] Garcia-Manrique J., Camas D., Gonzalez-Herrera A. (2017). Study of the stress intensity factor analysis through thickness: Methodological aspects. Fatigue Fract. Eng. Mater. Struct..

[8] Garcia-Manrique J., Camas-Peña D., Lopez-Martinez J., Gonzalez-Herrera A. (2018). Analysis of the stress intensity factor along the thickness: The concept of pivot node on straight crack fronts. Fatigue Fract. Eng. Mater. Struct..

[9] Urrego L.F., García-Beltrán O., Arzola N., Araque O. (2023). Mechanical Fracture of Aluminium Alloy (AA 2024-T4), Used in the Manufacture of a Bioproducts Plant. Metals.

[10] Chmelko V., Harakal’ M., Žlábek P., Margetin M., Ďurka R. (2022). Simulation of stress concentrations in notches. Metals.

[11] Braun M., Fischer C., Baumgartner J., Hecht M., Varfolomeev I. (2022). Fatigue Crack Initiation and Propagation Relation of Notched Specimens with Welded Joint Characteristics. Metals.

[12] Antunes F.V., Chegini A.G., Branco R., Camas D. (2015). A numerical study of plasticity induced crack closure under plane strain conditions. Int. J. Fatigue.

[13] Camas D., Antunes F., Moreno B., Gonzalez-Herrera A. (2019). Numerical analysis of the influence of the last cycle scheme on plasticity induced crack closure. Procedia Struct. Integr..

[14] Gardin C., Fiordalisi S., Sarrazin-Baudoux C., Gueguen M., Petit J. (2016). Numerical prediction of crack front shape during fatigue propagation considering plasticity-induced crack closure. Int. J. Fatigue.

[15] Marques B., Borrego L.P., Ferreira J.M., Antunes F.V., Branco R. (2019). A numerical analysis of fatigue crack closure using CTOD. Procedia Struct. Integr..

[16] Antunes F.V., Sousa T., Branco R., Correia L. (2015). Effect of crack closure on non-linear crack tip parameters. Int. J. Fatigue.

[17] Antunes F.V., Correia L., Camas D., Branco R. (2015). Effect of compressive loads on plasticity induced crack closure. Theor. Appl. Fract. Mech..

[18] Toribio J., Kharin V. (2011). Plasticity-induced crack closure: A contribution to the debate. Eur. J. Mech. A/Solids.

[19] Gardin C., Fiordalisi S., Sarrazin-Baudoux C., Petit J. (2016). Numerical simulation of fatigue plasticity-induced crack closure for through cracks with curved fronts. Eng. Fract. Mech..

[20] Calvín G., Escalero M., Zabala H., Muñiz-Calvente M. (2023). Effects of stress ratio on plasticity-induced crack closure through three-dimensional advanced numerical finite element models. Theor. Appl. Fract. Mech..

[21] Antunes F.V., Camas D., Correia L., Branco R. (2015). Finite element meshes for optimal modelling of plasticity induced crack closure. Eng. Fract. Mech..

[22] Liu J., Chen J., Sun Z., Zhang H., Yuan Q. (2023). A Study on Fatigue Crack Closure Associated with the Growth of Long Crack in a New Titanium Alloy. Metals.

[23] Borges M., Caldas M., Antunes F., Branco R., Prates P. (2020). Fatigue crack growth from notches: A numerical analysis. Appl. Sci..

[24] Ferreira F.F., Neto D.M., Jesus J.S., Prates P.A., Antunes F.V. (2020). Numerical prediction of the fatigue crack growth rate in SLM Ti-6Al-4V based on crack tip plastic strain. Metals.

[25] Neto D.M., Borges M.F., Sérgio E.R., Antunes F.V. (2022). Effect of Residual Stresses on Fatigue Crack Growth: A Numerical Study Based on Cumulative Plastic Strain at the Crack Tip. Materials.

[26] Mcclung R.C. (1991). Crack closure and plastic zone sizes in fatigue. Fatigue Fract. Eng. Mater. Struct..

[27] Sehitoglu H., Gall K., García A.M. (1996). Recent advances in fatigue crack growth modeling. Int. J. Fract..

[28] Ellyin F., Wu J. (1999). A numerical investigation on the effect of an overload on fatigue crack opening and closure behaviour. Fract. Eng. Mater. Struct..

[29] Wei L.-W., James M.N. (2000). A study of fatigue crack closure in polycarbonate CT specimens. Eng. Fract. Mech..

[30] Roychowdhury S., Dodds R.H. (2003). A numerical investigation of 3-D small-scale yielding fatigue crack growth. Eng. Fract. Mech..

[31] Subramanya H.Y., Viswanath S., Narasimhan R. (2005). A three-dimensional numerical study of mixed mode (I and II) crack tip fields in elastic-plastic solids. Int. J. Fract..

[32] Simandjuntak S., Alizadeh H., Smith D.J., Pavier M.J. (2006). Three dimensional finite element prediction of crack closure and fatigue crack growth rate for a corner crack. Int. J. Fatigue.

[33] She C., Guo W. (2007). Three-dimensional stress concentrations at elliptic holes in elastic isotropic plates subjected to tensile stress. Int. J. Fatigue.

[34] Proudhon H., Li J., Wang F., Roos A., Chiaruttini V., Forest S. (2016). 3D simulation of short fatigue crack propagation by finite element crystal plasticity and remeshing. Int. J. Fatigue.

[35] Zerbst U., Vormwald M., Pippan R., Gänser H.P., Sarrazin-Baudoux C., Madia M. (2016). About the fatigue crack propagation threshold of metals as a design criterion—A review. Eng. Fract. Mech..

[36] Lopez-Crespo C., Cruces A.S., Seitl S., Moreno B., Lopez-Crespo P. (2021). Estimation of the plastic zone in fatigue via micro-indentation. Materials.

[37] Palacios-Pineda L.M., Hernandez-Reséndiz J.E., Martínez-Romero O., Donado R.J.H., Tenorio-Quevedo J., Jiménez-Cedeño I.H., López-Vega C., Olvera-Trejo D., Elías-Zúñiga A. (2022). Study of the Evolution of the Plastic Zone and Residual Stress in a Notched T-6061 Aluminum Sample. Materials.

[38] Lin Z., Shang H., Gao H., Huang X. (2022). In Situ Measurement of the Strain Field at the Fatigue Crack Tip Based on Sub-Image Stitching and Matching DIC. Materials.

[39] Ajmal M., Lopez-Crespo C., Cruces A.S., Lopez-Crespo P. (2023). New Plastic Crack-Tip Opening Displacement Tool Based on Digital Image Correlation for Estimating the Fatigue-Crack-Growth Law on 316L Stainless Steel. Materials.

[40] Mokhtarishirazabad M., Lopez-Crespo P., Moreno B., Lopez-Moreno A., Zanganeh M. (2017). Optical and analytical investigation of overloads in biaxial fatigue cracks. Int. J. Fatigue.

[41] (2000). Standard Test Method for Measurement of Fatigue Crack Growth Rates.

[42] Antunes F.V., Serrano S., Branco R., Prates P. (2018). Fatigue crack growth in the 2050-T8 aluminium alloy. Int. J. Fatigue.

[43] Sato M., Moura L.S., Galvis A.F., Albuquerque E.L., Sollero P. (2019). Analysis of two-dimensional fatigue crack propagation in thin aluminum plates using the Paris law modified by a closure concept. Eng. Anal. Bound. Elements.

[44] Zerbst U., Madia M., Klinger C., Bettge D., Murakami Y. (2019). Defects as a root cause of fatigue failure of metallic components. I: Basic aspects. Eng. Fail. Anal..

[45] Ferreira S.E., de Castro J.T.P., Meggiolaro M.A., de Oliveira Miranda A.C. (2019). Crack closure effects on fatigue damage ahead of crack tips. Int. J. Fatigue.

[46] Oplt T., Hutar P., Pokorný P., Náhlík L., Chlup Z., Berto F. (2019). Effect of the free surface on the fatigue crack front curvature at high stress asymmetry. Int. J. Fatigue.

[47] Antunes F.V., Prates P.A., Camas D., Sarrazin-Baudoux C., Gardin C. (2019). Numerical prediction of fatigue threshold of metallic materials in vacuum. Eng. Fract. Mech..

[48] Hu Y., Cheng H., Yu J., Yao Z. (2020). An experimental study on crack closure induced by laser peening in pre-cracked aluminum alloy 2024-T351 and fatigue life extension. Int. J. Fatigue.

[49] Katinić M., Turk D., Konjatić P., Kozak D. (2021). Estimation of c* Integral for mismatched welded compact tension specimen. Materials.

[50] Bray J.W. (1990). Nunes, ASM—Properties and Selection: Nonferrous Alloys and Special-Purpose Materials. ASM Metals Handbook.

[51] Vasco-Olmo J.M., Camacho-Reyes A., Gonzales G.L.G., Díaz F. (2023). Investigation of Plasticity Effects on Growing Fatigue Cracks Using the CJP Model of Crack Tip Fields. Materials.

